# The Mannose Receptor: From Endocytic Receptor and Biomarker to Regulator of (Meta)Inflammation

**DOI:** 10.3389/fimmu.2021.765034

**Published:** 2021-10-14

**Authors:** Hendrik J. P. van der Zande, Dominik Nitsche, Laura Schlautmann, Bruno Guigas, Sven Burgdorf

**Affiliations:** ^1^ Department of Parasitology, Leiden University Medical Center, Leiden, Netherlands; ^2^ Cellular Immunology, Life and Medical Sciences (LIMES) Institute, University of Bonn, Bonn, Germany

**Keywords:** mannose receptor, sMR, metaflammation, macrophage, biomarker, immunometabolism, sCD206

## Abstract

The mannose receptor is a member of the C-type lectin (CLEC) family, which can bind and internalize a variety of endogenous and pathogen-associated ligands. Because of these properties, its role in endocytosis as well as antigen processing and presentation has been studied intensively. Recently, it became clear that the mannose receptor can directly influence the activation of various immune cells. Cell-bound mannose receptor expressed by antigen-presenting cells was indeed shown to drive activated T cells towards a tolerogenic phenotype. On the other hand, serum concentrations of a soluble form of the mannose receptor have been reported to be increased in patients suffering from a variety of inflammatory diseases and to correlate with severity of disease. Interestingly, we recently demonstrated that the soluble mannose receptor directly promotes macrophage proinflammatory activation and trigger metaflammation. In this review, we highlight the role of the mannose receptor and other CLECs in regulating the activation of immune cells and in shaping inflammatory responses.

## Introduction

**Figure 1:**
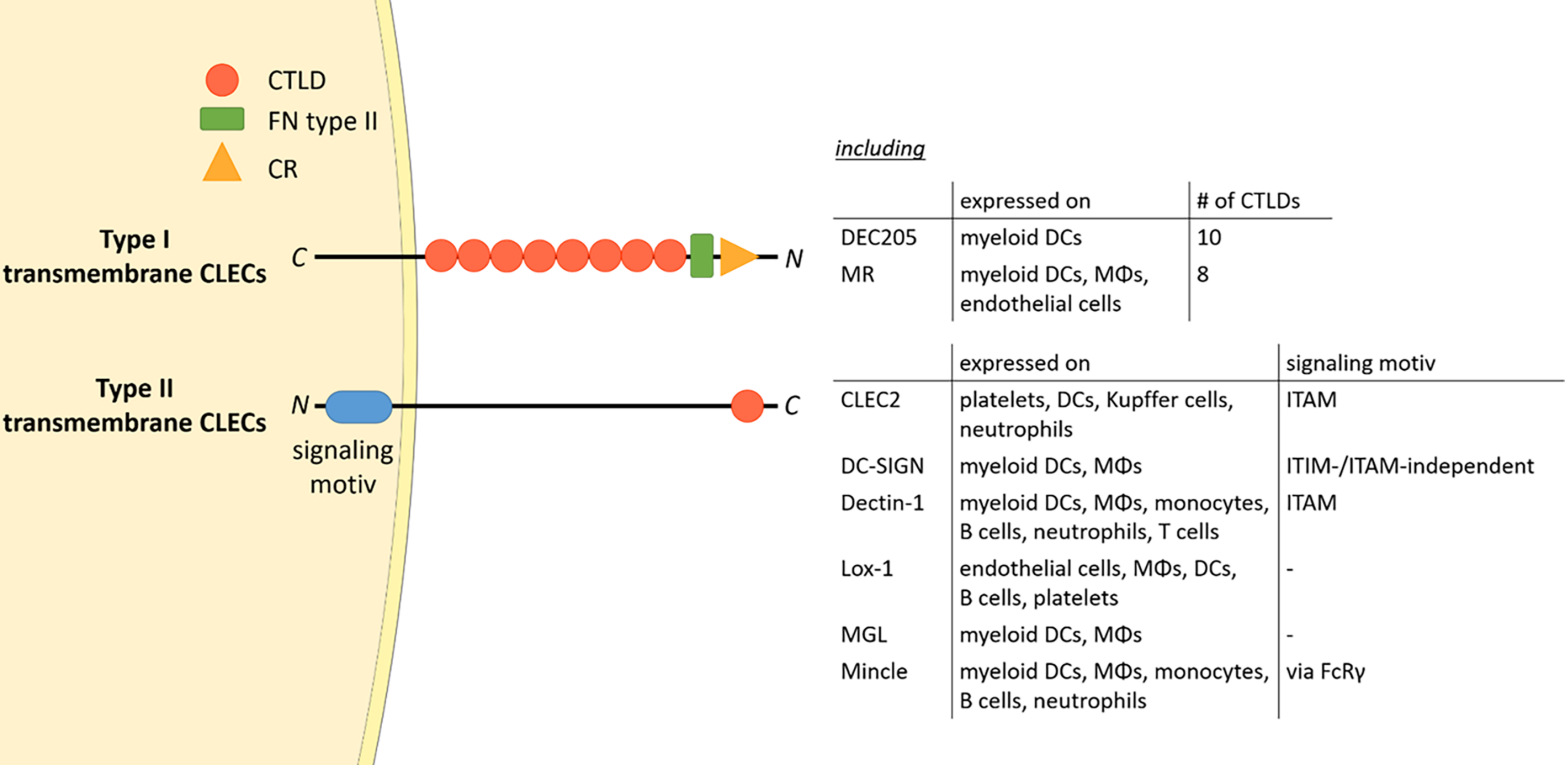
The CLEC family. Type I transmembrane CLECs typically contain multiple CTLDs at their extracellular region, whereas type II CLECs contain only one CLEC. All CLECs display individual expression patterns. Parts of the figure were created using templates from Servier Medical Art, which are licensed under a Creative Commons Attribution 3.0 Unported License; https://smart.servier.com. CTLD, C-type lectin domain; FN type II, fibronectin type II domain; CR, cysteine-rich domain; CLEC, C-type lectin; ITAM, immunoreceptor tyrosine-based activation motif; ITIM, immunoreceptor tyrosine-based inhibitory motif; DC, dendritic cell; MΦ, macrophage; FcRγ, Fc receptor gamma chain; C, C-terminus; N, N-terminus.

The mannose receptor (MR), also termed CD206, is a member of the C-type lectin (CLEC) family. Members of this family contain C-type lectin domains (CTLDs), which play an important function in ligand recognition. Typically, type I transmembrane CLECs contain multiple CTLDs at their extracellular region, whereas type II membrane CLECs only contain a single CLEC (**Figure 1**). In addition, type II transmembrane CLECs can bear signaling motives at their cytosolic tail (**Figure 1**).

The MR is mainly expressed by subpopulations of macrophages, immature dendritic cells (DCs) and endothelial cells (1, 2). Its expression level varies upon the situation and can be differentially regulated by cytokines (e.g. IL-10, IL-4, IL-13 and IFNγ), prostaglandins, LPS and the transcription factor PPAR-γ (3–7). Hence, MR expression is closely related to the activation status of the MR-expressing cell.

The MR encompasses a nearly 175 kDa type I transmembrane protein, consisting of an N-terminal cysteine-rich (CR) domain, a fibronectin (FN) type II domain, eight C-type lectin domains (CTLDs), a transmembrane region and a short cytosolic region. Similar to most other CLECs, a main feature of the MR is the recognition and internalization of specific ligands.

**Figure 2:**
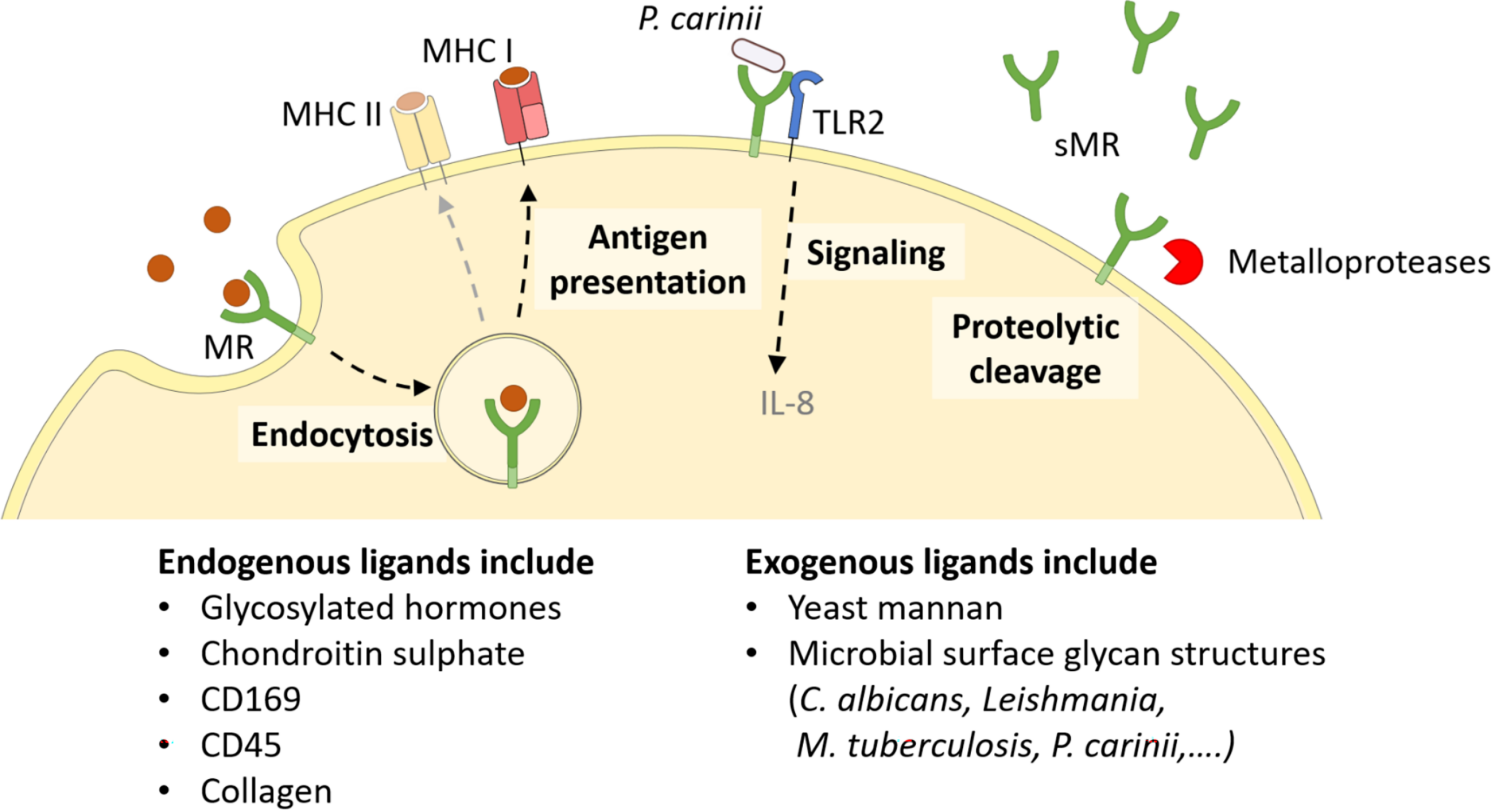
Cellular functions of the MR. The membrane-bound MR can recognize extracellular ligands, leading to their internalization. Endocytosed antigens are targeted into early endosomes, from which they are processed mainly for cross-presentation onto MHC I molecules and subsequent CD8^+^ T cell activation. Furthermore, the MR can assist other molecules in their signaling cascade, like enhanced TLR2 signaling after recognition of *P. carinii*. Finally, the MR can be shed by metalloproteases and released as a soluble form (sMR) in the extracellular space. MHC, major histocompatibility complex; MR, mannose receptor. Parts of the figure were created using templates from Servier Medical Art, which are licensed under a Creative Commons Attribution 3.0 Unported License; https://smart.servier.com.

Since every MR region has its own binding specificity, ligands can vary substantially in their molecular structure. The cysteine-rich domain mediates binding to sulphated sugars (8) including glycosylated hormones, chondroitin sulphate and sulphated Lewis^X^ and Lewis^A^ (9), but also specific proteins attached to sulphated glycostructures, such as CD169 and CD45 (10). With its fibronectin type II domain, the MR recognizes collagen (especially type I-IV) (2, 11), and mediates collagen internalization by macrophages and liver sinusoidal endothelial cells (2). Its CTLDs are responsible for the recognition of glycoconjugates. More precisely, CTLD4 binds to glycostructures with terminal mannose, fucose or N-Acetylglucosamine (GlcNAc) in a calcium-dependent fashion (12, 13). Since these sugar moieties are often exposed on microorganisms, the MR contributes to the clearance of a variety of infections, including *Candida albicans* (14), *Leishmania* (15, 16), *Mycobacterium tuberculosis* (17) and *Klebsiella pneumoniae* (18). Hence, the MR can bind to and internalize a variety of both endogenous ligands and pathogens (**Figure 2**).

Since the intracellular region of the MR lacks any known signaling domains, no MR-intrinsic signaling has been reported yet. Still, the presence of the MR has been linked to a direct induction of several target genes (19–21), probably because the MR might assist other receptors in their signaling cascade (**Figure 2**). For example, it has been demonstrated that the MR interacts with TLR2 after binding to *Pneumocystis carinii* and stimulates a TLR2-mediated signaling cascade (22). The molecular mechanisms enabling MR-mediated stimulation of signaling events, however, remain to be elucidated.

Apart from its membrane-bound form, the MR can also be proteolytically cleaved by metalloproteases and released into the extracellular space as a soluble form (sMR) (**Figure 2**) (23, 24). Consequently, sMR can be detected in the supernatant of MR-expressing cells and in the serum of mice and humans as a soluble protein. Additionally, a recent study also indicated the presence of sMR in extracellular vesicles (25).

As the sMR encompasses all extracellular regions of full length MR, preserving its main ligand binding properties (23, 24), this suggests that proteolytic cleavage must occur directly after the transmembrane region, in close proximity to the cell membrane. MR shedding occurs constitutively and levels of sMR correlate with the amount of total MR expressed in the cells (23). In addition to constitutive shedding in MR-expressing cells, MR shedding is specifically stimulated by fungal particles (*P. carinii, Candida albicans, Aspergillus fumigatus* and zymosan) and requires Dectin-1-mediated signaling (9, 26). However, whether this is due to activation of specific proteases involved in MR shedding or to other reasons has not been elucidated so far.

## The MR Mediates Antigen Uptake and Processing for Cross-Presentation

Due to its ligand binding capacities and its role in the clearance of multiple pathogens, the endocytic properties of the MR have been extensively studied. Under normal conditions, the MR localizes to the plasma membrane and in early endosomes, from where it is constantly recycled, even in the absence of ligands. Upon ligand binding, the MR is internalized in a clathrin-dependent fashion, a process mediated by the FENTLY motif in the cytoplasmic tail of the receptor. The di-aromatic YF motif is responsible for its intracellular trafficking into early endosomes (27).

Specific intracellular routing of MR-internalized antigens into early endosomes (28–33) was shown to have pronounced consequences for its role in antigen presentation. In fact, MR–internalized antigens are targeted towards a distinct non-degradative early endosome population, where they are rescued from lysosomal degradation and concomitant presentation on MHC II molecules (28). Mechanistically, the MR has been postulated to actively prevent the fusion of such early endosomes with lysosomes (34, 35). From this early endosomal compartment, MR-internalized antigens are predominantly processed for antigen presentation on MHC I molecules, a process called cross-presentation (**Figure 2**) (28, 36). Additionally, ligand binding to the MR induces its ubiquitination, which in turn contributes to the recruitment of the cross-presentation machinery. Interestingly, MR cross-linking using antigens conjugated to MR-specific antibodies can also induce lysosomal targeting and concomitant MHC II-restricted presentation of the internalized antigens (37–39). Additionally, antibody-mediated cross-linking of the MR has been demonstrated to activate an anti-inflammatory immunosuppressive program in antigen-presenting cells (APCs) (19), pointing out the possibility that ligand binding and receptor cross-linking might regulate the functional outcome of MR-mediated antigen recognition. The role of the MR in antigen uptake, processing and presentation, however, is extensively described elsewhere (40–42) and is not a central topic of this review.

In addition to its role in endocytosis, the MR has also been postulated to be involved in macrophage migration, as MR-deficient bone marrow-derived macrophages display increased migration independent of a CSF-1 gradient (43). Although the underlying molecular mechanisms remain to be identified, it is thinkable that these effects were mediated by MR-mediated interaction with collagen.

## Membrane-Bound MR on Antigen-Presenting Cells Induce T Cell Tolerance

Apart from its function in antigen recognition, internalization and processing for cross-presentation in APCs, the membrane-bound MR has been shown to directly regulate the function of other immune cells. Due to its association with antigen uptake and presentation, the MR became an attractive receptor in antigen targeting strategies. Such antigen targeting towards the MR has been linked to the induction of antigen-specific tolerance (44). In fact, in a mouse model of experimental autoimmune encephalomyelitis, injection of mannosylated myelin peptides surprisingly inhibited the onset of disease (45). Additionally, MR engagement on monocyte-derived DCs contributed to the induction of a regulatory phenotype (19, 46) and MR expression is mainly restricted to immunoregulatory cells, including tolerogenic DC subtypes, liver sinusoidal endothelial cells and alternatively activated macrophages (47, 48), for which the MR constitutes one of the main marker proteins.

**Figure 3:**
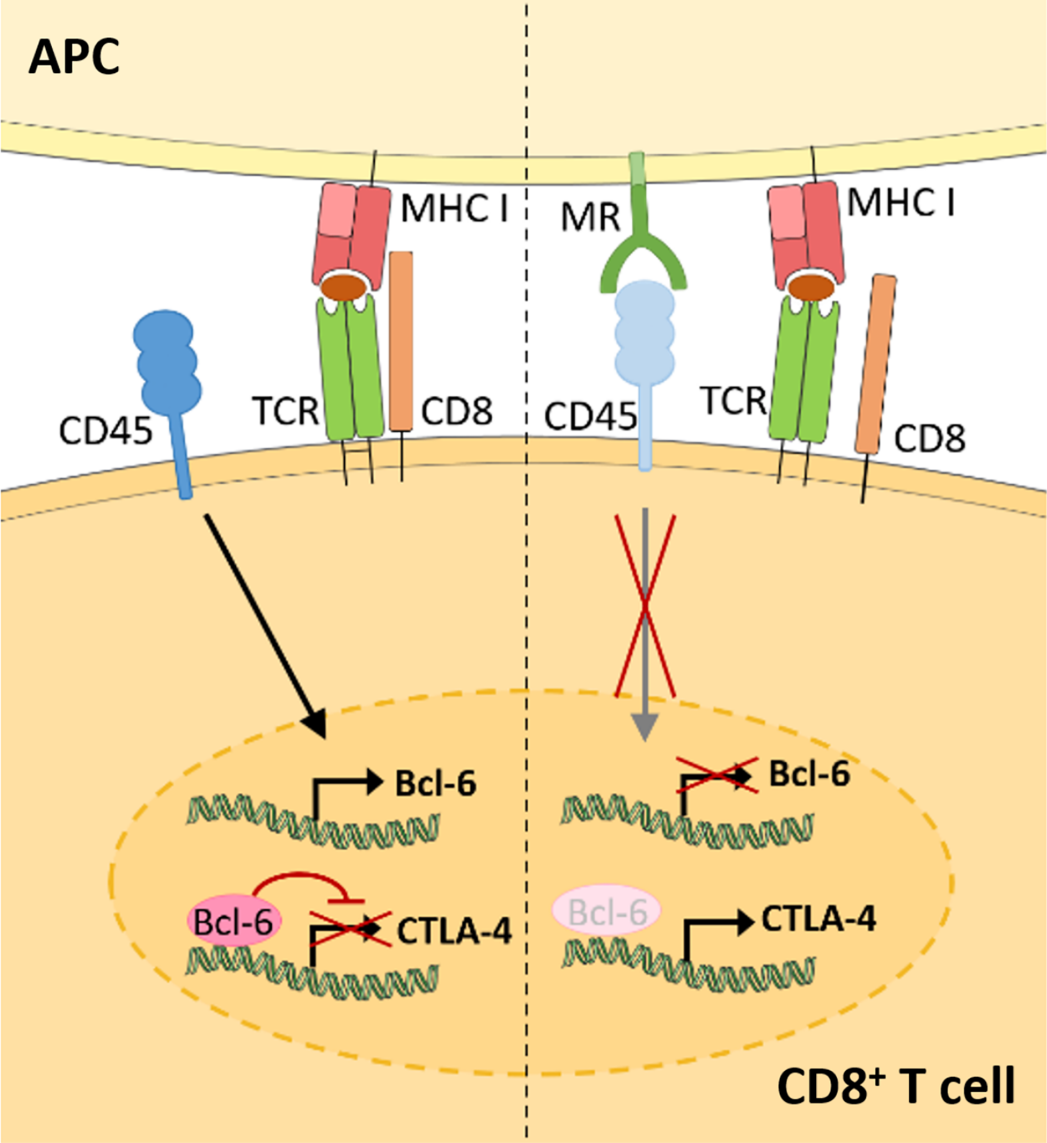
Membrane-bound MR on antigen presenting cells induces CD8^+^ T cell tolerance. Upon CD8^+^ T cell activation in the absence of the MR (left), expression of the transcriptional inhibitor Bcl-6 is induced. Bcl-6 binds to the CTLA-4 promoter and prevents its expression. During T cell activation in the presence of the MR (right), the MR on APCs interacts with CD45 on cytotoxic T cells. Such interaction prevents the upregulation of Bcl-6 and induces CTLA-4 expression and CD8^+^ T cell tolerance. APC, antigen presenting cell; MR, mannose receptor; TCR, T cell receptor. Parts of the figure were created using templates from Servier Medical Art, which are licensed under a Creative Commons Attribution 3.0 Unported License; https://smart.servier.com.

Recent advances demonstrate that the membrane-bound MR is not a mere marker for tolerogenic cells, but also plays an active role in the induction of T cell tolerance (**Figure 3**) (11). Indeed, CD8^+^ T cells activated by MR-expressing DCs displayed a clearly reduced cytotoxicity. This impaired T cell activation was mediated by a direct interaction of the membrane-bound MR on APCs with CD45 on T cells. CD45 is an immune cell-specific phosphatase which can be expressed as different isoforms depending on the immune cell subset. CD45 isoforms differ in the presence of the alternatively spliced exons A, B and C (49) and are frequently used to identify or distinguish bone marrow-derived immune cell subsets. Functionally, CD45 has been shown to play an important role in signaling mediated by the T and B cell receptors (50), whereas little is known about the role of CD45 in other immune cells. Importantly, the interaction of membrane-bound MR with CD45 on CD8^+^ T cells during T cell activation caused inhibition of its phosphatase activity, which resulted in T cell reprogramming and a significant upregulation of tolerance-associated genes. One of these genes encodes CTLA-4, which was mainly responsible for the impaired T cell cytotoxicity (11).

Surprisingly, MR-mediated inhibition of CD45 did not alter T cell receptor (TCR) signaling, as TCR-induced Lck activity, phosphorylation of ZAP70, LAT and ERK, intracellular calcium release and NFAT activation were not clearly influenced by the presence of the MR on the APC. However, transcription factor binding prediction analysis at the CTLA-4 promoter identified B-cell lymphoma 6 (Bcl-6), a transcription repressor normally involved in the differentiation of T follicular helper cells (51), as novel regulator of CTLA-4 expression. Using computational analyses, Electrophoretic Mobility Shift Assay (EMSA) and chromatin immunoprecipitation (ChIP) experiments, two Bcl-6 binding sites were identified within the CTLA-4 promoter. Indeed, Bcl-6 recruitment towards the CTLA-4 promoter prevented CTLA-4 transcription. Moreover, Bcl-6 expression was induced by CD45 phosphatase activity during T cell activation. Hence, MR-mediated inhibition of CD45 prevented the induction of Bcl-6 in activated T cells, eventually leading to the expression of CTLA-4 and the induction of T cell tolerance (**Figure 3**) (11), which was also confirmed *in vivo*. Injection of wild-type or MR-deficient DCs, that were previously transduced with OVA-expressing adenoviruses, resulted in an upregulation of CLTA-4 and impaired cytotoxic activity of antigen-specific T cells after priming by MR-expressing DCs (11). Accordingly, MR-deficient mice displayed a higher capacity of clearing an adenoviral infection when compared to wild-type mice (11), substantiating a regulatory function of the membrane-bound MR *in vivo*.

## sMR Correlates With Inflammatory Diseases and Induces Macrophage Activation

As mentioned above, the MR can be shed by proteolytic cleavage and released into the extracellular space. In contrast to the regulatory effect of the membrane-bound MR on T cell activation, the soluble form of MR has rather been associated with inflammation, as increased sMR serum levels have been observed in patients suffering from diverse inflammatory diseases.

First evidence for an association between sMR serum levels and disease progression came from a study in which increased sMR serum levels were observed in hospitalized patients when compared to a healthy control population (52). These differences were already pronounced in endocrinological and hematological patients, but became obvious in critically ill patients with sepsis and severe liver disease. Accordingly, the highest sMR concentrations were measured in the serum of patients from the intensive care unit. Similar observations were made in patients with liver cirrhosis, alcoholic liver disease and acute-on-chronic liver failure, a condition characterized by acute decompensation and organ failure following an extreme inflammatory response. Here, sMR concentrations were demonstrated to correlate with disease severity, portal hypertension, gut permeability, bacterial translocation and even mortality, displaying increased levels in non-survivors (53–58). Additionally, a modest but significant gender-independent correlation of sMR serum levels with age was observed (52).

Increased sMR levels were also observed in patients with a wide variety of inflammatory diseases, such as pulmonary tuberculosis (59), pulmonary fibrosis (60), multiple myeloma (61, 62), rheumatoid arthritis (63), chronic joint inflammation (64), pneumonia (65, 66), interstitial lung disease (67, 68) and gastric cancer (69). Strikingly, in these studies, sMR levels positively correlated with disease severity and mortality. As such, the sMR has been proposed as a new biomarker for inflammation (56, 57, 69–71). In fact, for several inflammatory diseases, including sepsis and pulmonary fibrosis, the sMR has even been suggested to be a better biomarker than those previously reported, such as sCD163, C-reactive protein or procalcitonin (60, 72). However, in all these studies, a functional role of the sMR in the onset of these inflammatory diseases has not been investigated so far.

The hypothesis of a putative functional role of sMR in inflammatory diseases is further supported by observations that MR-deficient mice are protected against inflammation-mediated renal injury in a mouse model of crescentic glomerulonephritis (CGN) (73). Macrophage infiltration in the kidney plays a dominant role in the pathophysiology of CGN (74, 75) and their phenotype is shaped by the kidney resident mesangial cells (MCs) (76). Interestingly, the protective effect of MR deficiency on CGN was associated with reduced macrophage infiltration in the kidney and impaired MC-mediated macrophage activation, as demonstrated by a reduction in both TNF secretion and phagocytosis-induced reactive oxygen species production. Although the potential contribution of sMR deficiency to CGN protection was not considered, these results provided the first evidence that MR may regulate proinflammatory activation of macrophages.

Importantly, a recent study demonstrated that the sMR can actually drive proinflammatory macrophage activation (77). sMR induced an inflammatory phenotype of both murine and human macrophages, as reflected by increased secretion of proinflammatory cytokines (TNF, IL–6, IL–12 and IL–1β) and a shift in cellular metabolism towards increased glycolysis (77), a hallmark of proinflammatory macrophage activation (78). In addition, RNAseq analyses also supported macrophage reprogramming towards an inflammatory phenotype (77), as the transcriptomic signature of sMR-treated macrophages displayed close similarities with the one of macrophages treated with TNF, prostaglandin E2 and the TLR2 ligand Pam3CSK4, a combination of stimuli used in a previous study to mimic a macrophage phenotype associated with chronic inflammation (79). Together, this demonstrates that sMR triggers an inflammatory response in macrophages.

**Figure 4:**
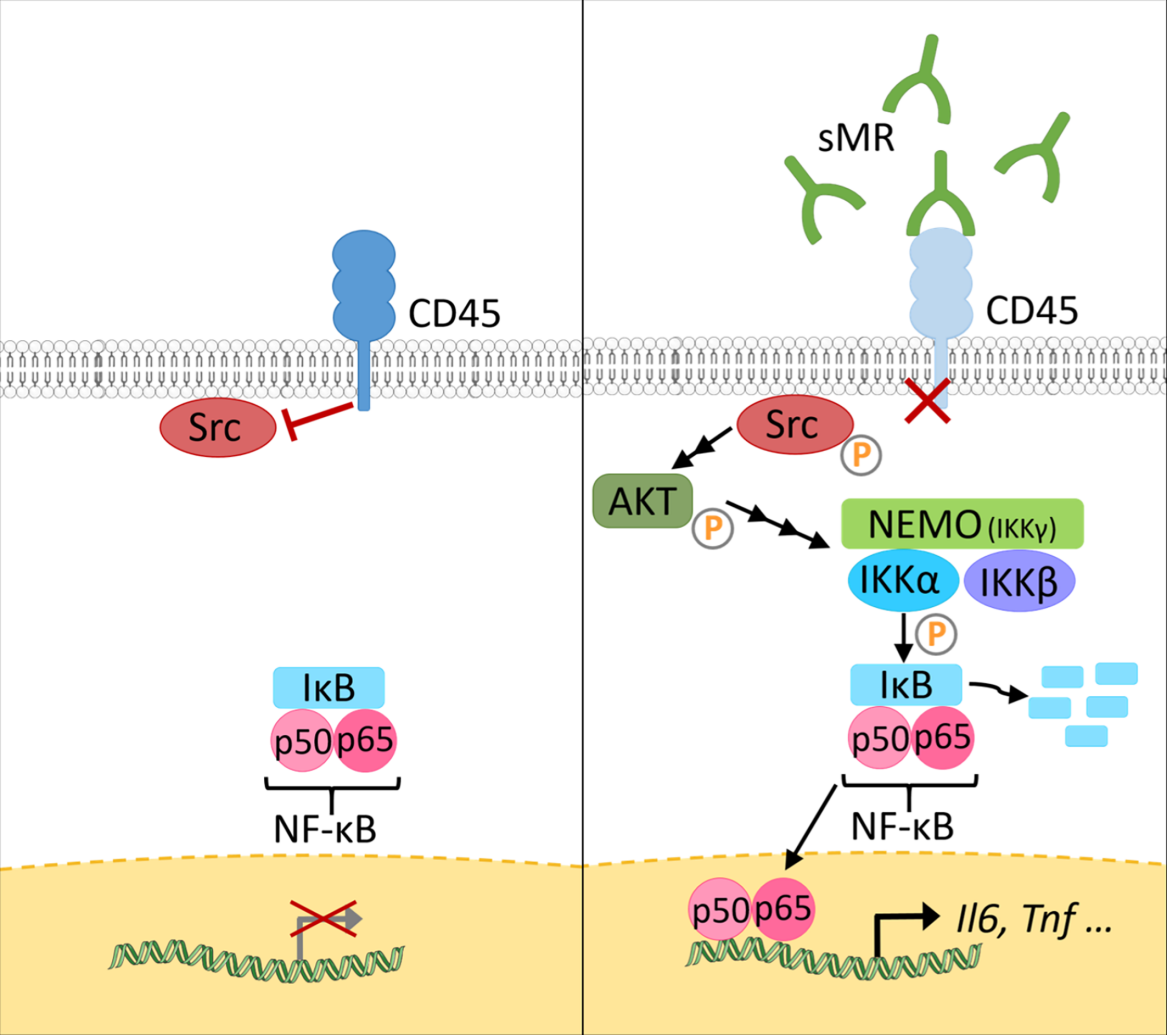
The sMR induces proinflammatory activation of macrophages. Under homeostatic conditions (left), CD45 in macrophages dephosphorylates Src. At increased sMR concentrations (right), binding of sMR to CD45 inhibits its phosphatase activity, leading to phosphorylation and activation of Src, which in turn activates an Akt/NF-κB pathway, causing macrophage reprogramming towards an inflammatory phenotype. sMR, soluble mannose receptor. Parts of the figure were created using templates from Servier Medical Art, which are licensed under a Creative Commons Attribution 3.0 Unported License; https://smart.servier.com.

At a mechanistic level, and similar to the effect of membrane-bound MR on T cells, sMR binds CD45 on macrophages, leading to an inhibition of CD45 phosphatase activity (77). Using specific inhibitors and siRNA-mediated downregulation of CD45, it was confirmed that sMR-induced proinflammatory macrophage activation was dependent on inhibition of CD45 (**Figure 4**). A screening for overrepresented transcription factor motifs in the promoter regions of all differentially expressed genes and identified NF-κB as the major transcription factor involved in sMR-induced macrophage activation. Accordingly, sMR treatment resulted in downregulation of IκBα, an inhibitor of NF-κB, and enhanced nuclear translocation of both NF-κB subunits p65 and p50 as well as recruitment of p65 to the TNF promotor. One of the known substrates of CD45 that has been associated with activation of NF-κB is Src, a kinase that is inactivated under homeostatic conditions by CD45-mediated dephosphorylation (80). Activated Src was shown to phosphorylate Akt (81), and both phosphorylated Src and Akt were reported to promote NF-κB activation (82–85). Using a combination of pharmacological and genetic tools, it was demonstrated that sMR-mediated inhibition of CD45 indeed resulted in a Src/Akt/NF-κB-mediated cellular reprogramming toward an inflammatory phenotype (**Figure 4**) (77).

## sMR Is a Novel Driver of Metaflammation

Proinflammatory macrophage accumulation in metabolic tissues is one of the hallmarks of obesity-induced metaflammation, a chronic state of low-grade inflammation that is triggering metabolic dysfunctions. Indeed, recruitment of CCR2^+^ monocytes to visceral white adipose tissue (WAT) and the liver promotes tissue inflammation, insulin resistance and impaired glucose homeostasis (86–89). This detrimental effect is believed to be mainly driven by monocyte differentiation into CD11c-expressing proinflammatory macrophages and enhanced production of TNF and IL-1β, leading to inhibition of canonical insulin signaling (90–92). Consequently, tissue-specific insulin resistance promotes ectopic lipid deposition and the development of hepatic steatosis, together contributing to whole-body insulin resistance. In support of this, genetic or pharmacological inhibition of CCR2-dependent monocyte recruitment to WAT and liver was shown to mitigate tissue inflammation and metabolic dysfunctions in obese mice (87, 88, 93).

**Figure 5:**
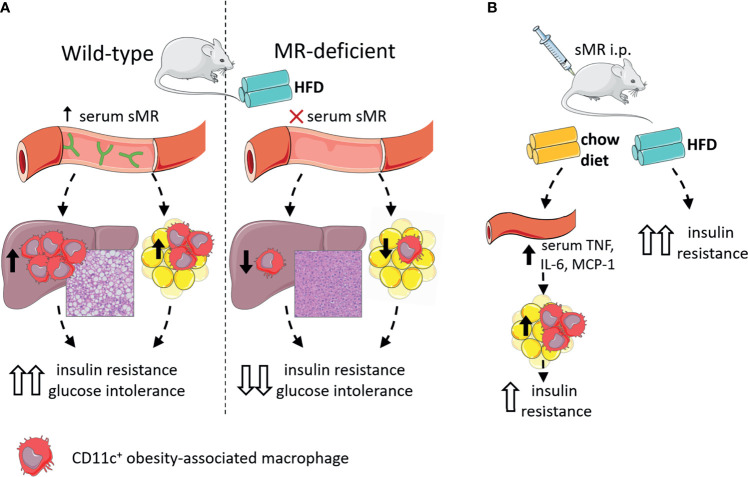
The sMR in metaflammation. **(A)** Wild-type mice on HFD (left) have high serum sMR, which is associated with increased hepatic steatosis, CD11c+ KCs and CD11c+ ATMs. Together, this is associated with increased insulin resistance and glucose intolerance. MR-deficient mice on HFD (right) have no serum sMR, which is associated with protection against hepatic steatosis, lower CD11c+ KCs and ATMs. Together, this is associated with lower insulin resistance and glucose intolerance. **(B)** sMR i.p. injections in mice on chow diet increased serum proinflammatory cytokines, associated with increased proinflammatory macrophages in adipose tissue, both associated with mild insulin resistance. sMR i.p. injection in mice on HFD increased insulin resistance. ATMs; adipose tissue macrophages; i.p., intraperitoneal; HFD, high-fat diet; KCs, Kupffer cells; sMR, soluble mannose receptor. Parts of the figure were created using templates from Servier Medical Art, which are licensed under a Creative Commons Attribution 3.0 Unported License; https://smart.servier.com.

In accordance with other inflammatory diseases discussed above, we recently reported that serum sMR levels were increased in high-fat diet (HFD)-fed obese mice and obese humans, and positively correlated with adiposity (77). Given that the sMR induces a pro-inflammatory phenotype in macrophages as described above and proinflammatory macrophages drive insulin resistance in metabolic tissues, these observations suggested the possibility that sMR-mediated proinflammatory macrophage activation in obesity may contribute to metabolic dysfunctions. Indeed, HFD-fed MR-deficient mice exhibited reduced numbers of CD11c-expressing obesity-associated macrophages in both WAT and liver, and were protected against hepatic steatosis, insulin resistance and glucose intolerance, independent of body weight changes (77) (**Figure 5A**). Of note, acute diphtheria toxin (DT)-mediated depletion of MR-expressing cells in obese CD206-DTR mice was also previously reported to improve whole-body glucose tolerance and insulin sensitivity when compared to wild-type mice (94), further substantiating a role for the MR in regulating metabolic homeostasis. In this study, the authors attributed the improved metabolic phenotype of these MR-deficient mice to increased proliferation and differentiation of adipocyte precursors in WAT secondary to downregulation of transforming growth factor (TGF)-β signaling pathway. However, since inflammatory macrophages and proinflammatory gene markers, especially *Tnf*, were also significantly reduced in WAT from obese MR-depleted mice, at least part of these observations could also be due to impaired MR-induced activation of macrophages.

More importantly, intraperitoneal administration of recombinant sMR to healthy lean mice acutely increased circulating proinflammatory cytokines (77), supporting that sMR can also trigger proinflammatory macrophage activation *in vivo*. As such, chronic treatment with sMR increased adipose tissue macrophage numbers, WAT expression of proinflammatory cytokines (**Figure 5B**) and reduced whole-body insulin sensitivity in lean mice, a detrimental metabolic effect that was even more pronounced when mice were concomitantly fed a HFD (77). These findings unequivocally identified the sMR as novel driver of macrophage activation and metaflammation.

## Other CLECs in the Regulation of Metaflammation

The proinflammatory effect of the MR on macrophages and its role in the development of obesity-induced metaflammation raises the question whether such properties are unique to the MR or rather a general feature of CLECs.

In general, CLECs can play a role in different kinds of immune responses. However, there are some striking similarities in the regulation of immune cell function between the MR and macrophage galactose-type lectin (MGL), another CLEC member that is also highly expressed on alternatively-activated macrophages. Similar to the MR, MGL lacks internal signaling motifs, but has been reported to enhance TLR2-mediated signaling (95). Additionally, membrane-bound MGL on APCs interacts with CD45 on T cells, inhibiting its phosphatase activity (96). Of note, in this study, the underlying molecular mechanisms seem to involve reduced T cell receptor signaling, and therefore differ slightly from MR-induced T cell tolerance. Nevertheless, MGL-induced inhibition of CD45 prevented effective activation of cytotoxic T cells (96). Strikingly, the immunometabolic phenotype of obese MGL-deficient mice resembles the phenotype of obese MR-deficient mice (97). Upon HFD feeding, both genotypes display reduced body weight gain, exclusively due to lower fat mass accumulation, protection against hepatic steatosis, and improved glucose tolerance and insulin sensitivity when compared to wild-type mice. Interestingly, these metabolic features were associated with reduced numbers of inflammatory macrophages in adipose tissue and a tissue-specific decreased in gene expression of *Ccl2* (MCP-1) and *Tnf* (77, 97). Although a potential interaction between MGL and CD45 on macrophages has not been investigated yet, it is tempting to speculate that MGL may inhibit CD45 phosphatase activity in macrophages, resulting in proinflammatory macrophage activation. The absence of such an interaction could potentially contribute to the protective immunometabolic phenotype of obese MGL-deficient mice. However, it is worth underlining that a soluble form of MGL has not been reported so far, suggesting that MGL-mediated effects, unlike those induced by the sMR, might require direct cell-cell interaction.

Another CLEC that has been involved in metaflammation is Dectin-1. As for MR-deficient mice, Dectin-1-deficient mice are protected from HFD-induced obesity (98). Dectin-1 expression was upregulated in WAT from obese mice and humans, and associated with proinflammatory adipose tissue macrophages (ATMs). Accordingly, treatment with a Dectin-1 antagonist improved insulin sensitivity in obese mice and reduced adipose tissue CD11c^+^ obesity-associated macrophages, while treatment with a Dectin-1 agonist did the opposite. However, since Dectin-1 ligation induces cellular signaling that directly leads to activation of NF-κB (99), it is likely that Dectin-1 promotes metaflammation through a different molecular mechanism than the MR. Nevertheless, increased metalloprotease-mediated MR shedding in response to *Candida albicans* and β-glucan particles was dependent on Dectin-1 and its intracellular signaling pathway (9), which offers the possibility that the immunometabolic phenotype of obese Dectin-1-deficient mice may in part be explained by reduced sMR production.

Macrophage-inducible C-type lectin (Mincle) has also been associated with a variety of inflammatory diseases, such as rheumatoid arthritis, allergic contact dermatitis, hepatitis and diet-induced obesity (100–103). Macrophage expression of Mincle was shown to be induced by saturated fatty acids and macrophage-adipocyte interactions (103). Accordingly, WAT Mincle expression was localized to crown-like structures of macrophages surrounding dying adipocytes during obesity (104). Although Mincle-deficient mice display similar weight gain compared to wild-type mice upon HFD feeding, obesity-induced crown-like structures, hepatic steatosis and whole-body insulin resistance and glucose intolerance are significantly mitigated when compared to wild-type mice (104, 105). As Mincle ligation induces FcRγ-mediated signaling, eventually resulting in activation of NF-κB in macrophages (99), Mincle-mediated macrophage activation is probably occurring *via* distinct molecular pathways, independent of CD45 and the MR.

Of note, there is a variety of other CLECs that were associated with metaflammation or chronic inflammatory diseases for which the mechanistic underpinnings are poorly defined. For example, expression of the lectin-like oxidatively-modified low-density lipoprotein (Ox-LDL) receptor (LOX–1) – also named CLEC8A – is increased in visceral WAT of HFD-fed obese mice (106). Obese LOX-1-deficient mice display reduced HFD-induced CCL2/MCP-1, macrophage inflammatory protein-1α (MIP-1a) and IL-6 expression in WAT, suggesting a role for LOX-1 in regulating adipose tissue inflammation. Interestingly, LOX-1 is expressed on endothelial cells (107) and human macrophages (108), and similar to the MR, it can be proteolytically cleaved to release a soluble form (109, 110). Although no functional role has been described for soluble LOX-1 to date, it is known that cleavage of LOX-1 is triggered by the pro-inflammatory factors oxLDL, C-reactive protein, TNF, IL-8 and IL-18 and regulated by membrane cholesterol (111–114). Its cleavage is mediated *via* serine proteases that have been shown to be upregulated during obesity, potentially increasing bioavailability of soluble LOX-1 in these conditions (115). Interestingly, soluble LOX-1 serum levels have been shown to be correlated with the occurrence and severity of a variety of inflammatory cardiovascular diseases, including stroke, arteriosclerosis and acute coronary syndrome (116–122). Whether soluble LOX-1 is merely a biomarker for these diseases, or might be functionally involved in disease progression, remains to be identified.

The Dendritic Cell-Specific Intercellular adhesion molecule-3-Grabbing Non-integrin (DC-SIGN) - also termed CD209 or CLEC4L - is increased on monocyte-derived dendritic cells (Mo-DCs) from post-menopausal type 2 diabetic obese women, which is thought to modulate their adhesion capacity to vascular cell walls and migration to peripheral tissues (123). Besides this association with obesity, there is limited data on the putative role of DC-SIGN in the context of metaflammation. Similar to the MR, DC-SIGN can be detected as soluble form (sDC-SIGN) in serum (124) but its functions remain also largely unknown and would definitely require dedicated studies.

In contrast to the abovementioned detrimental roles of several CLECs in the context of obesity-induced metabolic dysfunctions, *in vivo* overexpression or administration of a soluble form of CLEC2 improved hepatic steatosis, hepatic fatty acid oxidation and whole-body glucose tolerance (125, 126). CLEC2 is expressed on platelets, dendritic cells, neutrophils and Kupffer cells, and its soluble form induced alternative activation of hepatic Kupffer cells, a feature that was postulated to drive the metabolic benefits, although this remains to be firmly established.

In conclusion, the proinflammatory effects of sMR and its role in obesity-induced metaflammation are not a general feature of CLECs. However, while the number of studies is limited, different CLECs have been linked to metaflammation, with the majority playing detrimental roles in the control of insulin sensitivity. Although some homogeneity in molecular mechanisms might exist (*e.g.* immunometabolic phenotypes of MGL-deficient and MR-deficient mice), other CLECs likely aggravate metabolic dysfunctions independent of interaction with CD45 on macrophages. As the conclusions from these studies were mostly drawn using whole-body knockout mice, future studies using conditional knockout models are warranted to identify the cellular source and underlying molecular mechanisms responsible for CLEC-mediated control of metabolic homeostasis.

## Discussion and Further Perspectives

Since the MR lacks signaling motifs, it was generally assumed that it functions as a mere endocytic receptor, internalizing extracellular material for clearance and antigen presentation. Recent advances have made clear that the MR can actively shape immune responses by directly regulating immune cell activity (11, 73, 77). Until now, the membrane-bound MR has been shown to induce T cell tolerance, whereas the sMR stimulates an inflammatory response in macrophages, both *via* inhibition of CD45. However, it remains unclear whether these observed differences are merely due to a distinct cell type-dependent role of CD45 or rather to different effects of the soluble *versus* membrane-bound MR. As membrane-bound MR could cross-link CD45 or alter its composition and clustering in the cell-membrane, a different response in terms of immune cell activation compared to its soluble form could be possible. First indications suggested that sMR might also promote T cell tolerance (11), pointing out that the recipient cell might determine the MR-induced effects rather than the form of MR interacting with the cells. Future studies will have to validate this hypothesis and show whether interaction of macrophages with membrane-bound MR also results in the induction of an inflammatory response. Similarly, the exact role of other soluble CLEC receptors, such as LOX-1, needs to be investigated carefully.

Moreover, it remains unclear whether the MR also influences the functionality of other immune cells, like CD4^+^ T cells, DCs and B cells. Since all these cells express CD45, a similar regulation by interaction with MR could be possible. Therefore, the identification of the CD45 isoforms interacting with the MR needs to be monitored carefully, since these isoforms differ substantially depending on the cell type and inflammatory status.

Another important open question regarding increased sMR serum concentrations during inflammation is the identification of its source. As mentioned above, the MR is mainly expressed by macrophages, DCs and endothelial cells (1, 2). During metaflammation, we observed increased MR expression in liver and adipose tissue but not spleen, in particular in macrophages and liver sinusoidal endothelial cells (77). As such, it can be expected that these cells are responsible for increased sMR production, resulting in enhanced local and systemic sMR concentrations and in macrophage-mediated metaflammation. Since the expression of the MR is directly regulated by PPARγ (7), and free fatty acid-activated PPARγ signaling is upregulated in lipid-associated macrophages during obesity (127), this transcription factor could be one of the key players in the regulation of MR expression and shedding. It is thus tempting to speculate that increased MR expression and hence sMR serum levels might be a result of metaflammation-associated activation of PPARγ in macrophages. In addition, MR is constitutively cleaved by yet unidentified metalloproteases (23, 24). Since obesity was shown to alter the metalloprotease expression profiles of adipose tissue and liver (128–130), it needs to be investigated whether obesity-induced metalloprotease expression in metabolic tissues may increase MR shedding.

Additionally, the correlation of sMR serum concentrations with the inflammatory status of various human populations should be monitored carefully and in an unbiased fashion, using a large and representative cohort not selected for specific inflammatory conditions. Naturally, future studies should address whether sMR-mediated activation of macrophages plays a functional role in the onset and progression of such conditions. In order to experimentally address this putative function of the sMR in different diseases, the availability of reliable methods to quantify sMR serum levels is a prerequisite. For this purpose, ELISA-based methods to quantify human and mouse sMR are commercially available. Such ELISA kits have been reported manyfold to reliably determine sMR levels in human serum (52). However, studies reporting sMR levels in murine sera are rare, which might be explained by a lack of reliability of the available products. Indeed, we recently developed a method based on immunoprecipitation and fluorimetry to monitor murine sMR serum levels in the context of metaflammation (77), as we obtained false positive detection of sMR serum levels from MR-deficient mice using a commercially available ELISA kit. However, since this technique is elaborate and time-consuming, the establishment of a reliable ELISA is of interest to monitor sMR in mouse serum in future investigations.

In conclusion, should the sMR be confirmed to contribute to the induction of inflammation in a broad spectrum of diseases, it would definitely constitute a potential target for therapeutic intervention. As such, approaches aimed at reducing, eliminating or inactivating sMR might reduce macrophage activation and could contribute to mitigation of disease. In addition, the molecular mechanisms leading to increased sMR serum concentrations are also of great interest, as these could provide additional leads for therapeutic interventions.

## Author Contributions

HZ, DN, and LS: writing and conceptualization. BG and SB writing, conceptualization, and supervision. All authors contributed to the article and approved the submitted version.

## Funding

This work is funded by the Deutsche Forschungsgemeinschaft (DFG, German Research Foundation) - SFB1454 (project number 432325352 to SB) under Germany´s Excellence Strategy EXC2151 (project number 390873048 to SB), the NWO Graduate School Program (022.006.010 to HZ), an EFSD/Lilly Research Grant Fellowship from the European Federation for the Study of Diabetes (to BG) and the Dutch Organization for Scientific Research (ZonMW TOP Grant 91214131 to BG).

## Conflict of Interest

The authors declare that the research was conducted in the absence of any commercial or financial relationships that could be construed as a potential conflict of interest.

## Publisher’s Note

All claims expressed in this article are solely those of the authors and do not necessarily represent those of their affiliated organizations, or those of the publisher, the editors and the reviewers. Any product that may be evaluated in this article, or claim that may be made by its manufacturer, is not guaranteed or endorsed by the publisher.
